# Assessing controls on mass budget and surface velocity variations of glaciers in Western Himalaya

**DOI:** 10.1038/s41598-018-27014-y

**Published:** 2018-06-11

**Authors:** Shashank Bhushan, Tajdarul H. Syed, Anthony A. Arendt, Anil V. Kulkarni, Debanjan Sinha

**Affiliations:** 1 Department of Applied Geology, Indian Institute of Technology (ISM), Dhanbad, India; 2 Applied Physics Laboratory, University of Washington, Seattle, USA; 3 eScience Institute, University of Washington, Seattle, USA; 4 Divecha Centre for Climate Change, Indian Institute of Science, Bengaluru, India

## Abstract

This study analyses spatially resolved estimates of mass budget and surface velocity of glaciers in the Zanskar Basin of Western Himalaya in the context of varying debris cover, glacier hypsometry and orientation. The regional glacier mass budget for the period of 1999–2014 is −0.38 ± 0.09 m w.e./a. Individual mass budgets of 10 major glaciers in the study area varied between −0.13 ± 0.07 and −0.66 ± 0.09 m w.e./a. Elevation changes on debris-covered ice are considerably less negative than over clean ice. At the same time, glaciers having >20% of their area covered by debris have more negative glacier-wide mass budgets than those with <20% debris cover. This paradox is likely explained by the comparatively larger ablation area of extensively debris-covered glaciers compared to clean-ice glaciers, as indicated by hypsometric analysis. Additionally, surface velocities computed for the 2013–14 period reveal near stagnant debris-covered snouts but dynamically active main trunks, with maximum recorded velocity of individual glaciers ranging between ~50 ± 5.58 and ~90 ± 5.58 m/a. The stagnant debris-covered extent, which varies from glacier-to-glacier, are also characterized by ice cliffs and melt ponds that appreciably increase the overall surface melting of debris-covered areas.

## Introduction

Significant mass loss is evident over major glacierized regions in High Mountain Asia^1^. The only exceptions are the Karakoram range and the western Kunlun Shan where glaciers show a more or less balanced mass budget^2–4^. Most of these estimates were obtained from studies conducted over large spatial domains and those conducted on basin or catchment scales are mostly limited to certain sites only (for e.g. Karakoram^5,6^, Tien Shan^7,8^, Nepal^9–11^, Lahul and Spiti^12^). While site-specific studies reveal important information about the influence of local factors such as glacier orientation, hypsometry, morphology and surrounding topography on glacier dynamics, regional scale studies provide valuable insights into the climatic conditions driving glacier mass changes and thus present a larger picture of global impacts on glacier dynamics as a whole.

Previous studies have indicated significant deviations in the mass budgets of glaciers, even within a single basin, which is controlled by different factors such as glacier hypsometry, orientation, extent of debris cover and surface velocity^12–14^. Detailed characterization of these controls on glacier mass budget at basin/catchment scales is crucial for ascertaining the status of glaciers^6,8^ and for characterizing heterogeneity in glacier dynamics^15,16^ and mass budget^5,11^, which have important implications for calibration of hydrologic^17^ and glaciological^18^ models and assessment of melt-water contributions to river discharge^19,20^.

Among the above mentioned controlling factors, the effect of debris cover is still actively debated^1,21–23^. In general, thick debris cover can appreciably reduce glacier melting rates^9,24,25^ by acting as an insulating layer^26^. Further, debris-covered glaciers can exhibit significant heterogeneity in elevation change patterns (e.g. closely spaced regions showing conspicuous positive and negative elevation change^9^). However, using a statistical approach on the above-mentioned factors, Salerno *et al*.^14^ concluded that glacier mass budget is primarily dependent on surface gradient and practically independent of debris-cover percentage.

**Figure 1:**
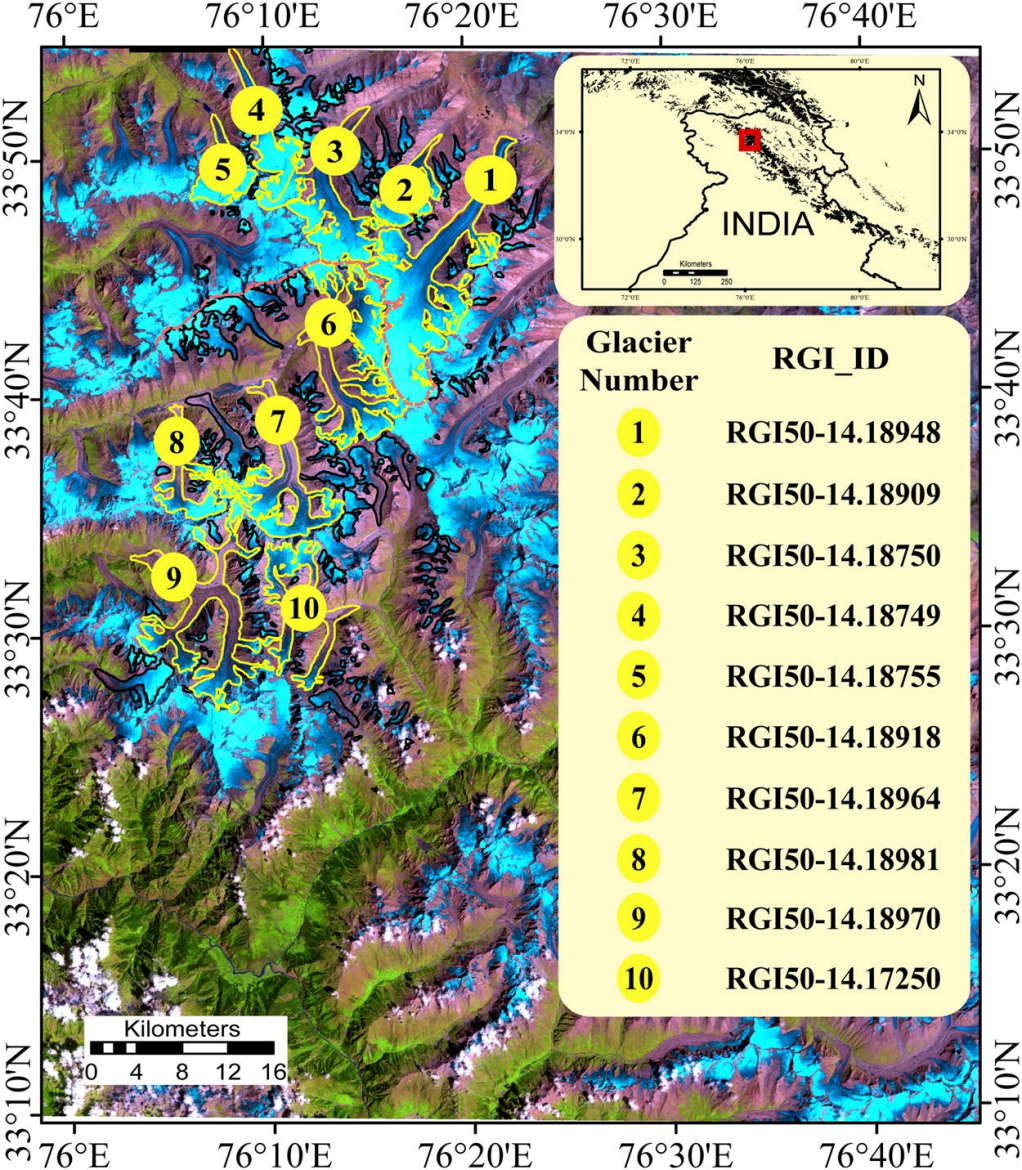
False Colour Composite of Landsat image acquired on 17^th^ August, 1999 showing the boundaries of glaciers (black polygons) in the study area. Glaciers with an area greater than 10 km^2^ are enumerated and highlighted. The location of topographic divide is indicated by the dashed red line. Insets show the location of the study site in context of the physical map of India and the RGI IDs of the highlighted glaciers. (The map was generated in ArcGis 9.3 Master Lab Kit and post processed using Adobe Illustrator Creative Cloud).

The Zanskar Basin is situated in the transition zone between the Karakoram Range and Western Himalaya (Fig. 1) and thus has significantly different climatic conditions when compared to other Himalayan regions. The Zanskar Basin lies in the rain shadow area of the Himalayan range^27^ with most of the precipitation occurring in winter as a result of Mediterranean influences of the mid-latitude westerlies (MLW)^28^. Glaciers in Zanskar Basin have been retreating with an overall increase in debris-covered area for the past three decades^27^. However, the most recent mass budget analysis of six selected glaciers in the Zanskar Basin including the Drung Drung Glacier, using the Equilibrium Line Altitude (ELA) and Accumulation Area Ratio (AAR) method, reported an overall gain in volume (~206.5 km^3^a^−1^) during the period of 2000–2010^29^. This contradicts almost all previous estimates, which have reported significant glacier mass loss in the Western Himalaya^3,4,30^.

In this study, mass budgets for glaciers in the Zanskar Basin, spread over an area of ~437 km^2^, are computed for the period 1999–2014. Elevation changes are calculated from Digital Elevation Models (DEMs) derived from Cartosat-1 stereo pairs and Shuttle Radar Topographical Mission (SRTM) C Band DEMs. Also analysed are estimates of glacier surface velocity, computed using feature tracking techniques on Landsat 8 images, for the period 2013–14. Subsequently, variations in elevation changes are investigated in the context of supraglacial debris cover, surface velocity, hypsometry and glacier orientation in order to understand the mechanisms influencing the observed variations.

## Data

In this study, we use the Shuttle Radar Topographical Mission (SRTM) Digital Elevation Model (DEM) (1 arc second, version 3), computed for February 2000 using two C Band radar antenna with a spatial resolution of approximately 30 m, as our reference DEM^31^. The C band interferometric signal has been reported to penetrate snow/ice, yielding discrepancies in the surface elevation of ice caps and glaciers^32,33^. Assuming full penetration of C band signal, ice surface represented by STRM DEM in ablation areas can be approximated to be the surface representative of the end of ablation season in 1999^6,34^. However, the C band radar penetrates deeper into the snow layers in accumulation areas, resulting in a low bias in elevation values^33^. In this study the required penetration corrections over snow and ice facies are performed using the penetration estimates for the Jammu & Kashmir region mentioned in Kääb *et al*.^2^.

Two pairs of Cartosat-1 stereo images (Path/Row: 513/242, 513/243; acquired on 11^th^ July 2014) are used to derive the DEM of the study area for the second investigation timeframe. The Cartosat-1 satellite was launched in 2005 by Indian Space Research Organization (ISRO) and has two panchromatic cameras with stereo capabilities in the along-track direction. The spatial resolution of the sensor is 2.5 m with one scene covering a swath of 25 × 25 km. With a base to height ratio of 0.62 and a radiometric resolution of 10 bits, Cartosat-1 scenes have been effectively used to generate DEMs for glaciological applications^5,6,35^.

Landsat 8 OLI scenes from 14^th^ July 2013 and 18^th^ August 2014 are used to compute annual surface velocity while a Landsat ETM+ scene from 17^th^ August 1999 is used to delineate different glacial facies (ice, snow and debris-covered areas) for applying C Band penetration correction and other analysis. The same ETM+ scene was used in Kääb *et al*.^2^ for delineating glacier facies and to estimate the C Band penetration in different glacier facies over the Jammu & Kashmir region.

Glacier polygons from the Randolph Glacier Inventory (RGI 6.0^36^) are used to define glacier extents in the region. The RGI polygons for this area were derived from satellite imagery of October 2000 and the overall quality of the polygons was found to be of a good standard. Slight discrepancies in extent, especially near the debris-covered snout, is rectified on the basis of the elevation change (Cartosat-1 minus SRTM) maps obtained in this study, following Vijay and Braun^12^.

## Methodology

### DEM Generation and Mass Budget Computation

Cartosat-1 DEMs are generated using the rational function model in Rolta Geomatica 15. Around 100, precise and well distributed, tie points are collected for both the stereo pairs with an overall RMSE less than 1 m in ground coordinates. An optimum spatial distribution of tie points is important for ensuring overlap between the two scenes of the stereo pairs. The math model of the rational function is built upon the Rational Polynomial Coefficients (RPCs), which are required to establish the mathematical relationship between the ground coordinates and image pixels. The parameters used in extracting the DEMs are similar to those used in Berthier *et al*.^37^, with the type of terrain set as ‘*Mountainous*’ and the DEM detail set to ‘*Low*’. The output DEMs are extracted at 10 m resolution in the UTM 43 N posting. Subsequently, the Cartosat-1 DEM is planimetrically coregistered with the reference DEM (SRTM) to ensure perfect overlap over corresponding pixels (Supplementary Section 1). After that, the SRTM DEM is subtracted from the Cartosat-1 DEM to obtain the elevation change map. Outliers over glacierized areas are removed using a sigmoidal function (Supplementary Section 2) and then corrected for penetration of C Band signal using the estimates mentioned in Kaab *et al*.^2^ (Supplementary Section 3) (See Supplementary Fig. S1). The resultant data gaps in the elevation difference map are filled by ordinary kriging method^38^ (Supplementary Section 2).

Even though we present region-wide estimates of elevation change and mass budget of the study area (comprising of 236 glaciers), we focus on a subset of it for detailed analysis. For example, we only consider glaciers with an area >1 km^2^, which is 43 in number, for statistical analysis of relationships between glacier mass budget and factors controlling it. This is because, mass budget of glaciers with smaller area and limited elevation range is largely influenced by their overall elevation (See Supplementary Section 4). However, for a detailed assessment of variations in glacier-wide elevation change we concentrate on the 10 largest glaciers which cover a major portion (~68%) of the total glaciated area in the study site.

The glacier-wide elevation change is obtained by computing the area weighted average of the median elevation change in each 50 m elevation bin. Subsequently, the final mass budget estimate is obtained from the elevation change values by using a density value of 850 ± 60 kg/m^3^ ^39^. Regional estimates of glacier-wide elevation change and mass budget are obtained by computing the area weighted average of glacier-wide elevation change and mass budget values of the 236 glaciers identified in the study area respectively. The uncertainty in mass budget estimates is computed by taking into consideration the uncertainty involved in DEM differencing (See Supplementary Fig. S2 and Supplementary Table S1) and the uncertainty associated with the penetration and density values used in our analysis (See Supplementary Section 3).

In order to assess the hypsometric controls on glacier mass budget, glaciers within the study area are classified using a Hypsometric index (HI) calculated by (1)^40^.

1 $$HI=\frac{{H}_{max}-{H}_{med}}{{H}_{med}-{H}_{min}}$$

where, *H*_*max*_ is the maximum glacier elevation, *H*_*min*_ is the minimum glacier elevation and *H*_*med*_ is the median glacier elevation. If the HI value obtained is less than 1, then HI is recomputed as the negative inverse of the originally obtained HI. Accordingly, the glaciers are categorized into *very top heavy* (HI < −1.5), *top heavy* (−1.5 < HI < −1.2), *equidimensional* (−1.2 < HI < 1.2), *bottom heavy* (1.2 < HI < 1.5) and *very bottom heavy* (HI >1.5) glaciers. For instance, a HI value of 2.87 (Boama Glacier (glacier number 9)), signifies that the upper 50% of glacier area spans an elevation range that is 2.87 times larger than that of the lower 50% of the glacier area,. thereby, making the glacier very bottom heavy since the area distribution is strongly skewed towards the lower elevation range^41^.

### Velocity Estimates

Glacier surface velocities exert important controls on its mass budget. Variations in velocity are manifested as changes in submergence (emergence) patterns of ice flux in the accumulation (ablation) areas, thus causing dynamic feedbacks on glacier mass budget^42,43^. Therefore, in order to facilitate our analysis of glacier mass budget, we derive surface velocity of the glaciers using the COSI-CORR algorithm^44^ (http://www.tectonics.caltech.edu/slip_history/spot_coseis/download_software.html) on panchromatic bands of Landsat 8 images. The two images in consideration were acquired at the end of ablation season with the time interval between acquisition being close to a year (Section 2.1). Feature tracking is accomplished using an initial window size of 64 × 64 scaled down to a final size of 32 × 32 with a step size of 2. Subsequently, displacement maps in East-West (x) and North-South (y) direction with a map of their corresponding Signal to Noise Ratio (SNR) are obtained. All pixels having SNR less than 0.9 are eliminated from future analysis and a median filter is run on the resulting data to filter out pixels which show a local reversal in x and y values^35^. Data gaps are filled using the mean of the surrounding 8 pixels. The resulting displacements in the x and y direction are summed up as per Eulerian norms after verifying proper alignment of the displacement vectors within the glacier margin. The final surface velocity map is computed from the displacement maps taking into consideration a time interval of one year.

Ideally, stable (ice-free) areas should be static (showing no displacement). However, due to limitations in the feature tracking algorithm used here, even the stable areas are observed to have certain displacement. Hence, uncertainty in the velocity estimates is quantified as the 68.3^rd^ percentile of the velocity over stable areas in the entire scene (See Supplementary Fig. S3 and Supplementary Table S2).

### Data Availability

The datasets generated during and/or analysed during the current study are available from the corresponding author on reasonable request.

## Results and Discussion

Results reveal a region-wide elevation change of −0.45 ± 0.1 m/a amounting to a specific mass budget of −0.38 ± 0.09 m w.e./a. This estimate corresponds well with the estimates from Gardner *et al*.^30^ and Brun *et al*.^4^ (−0.45 ± 0.14 m w.e./a and −0.37 ± 0.09 m w.e./a for the periods of 2003–2009 and 2000–2016 respectively). The mass budget of glaciers in the nearby Lahul and Spiti region^12^ also show a similar trend but with greater magnitude (−0.53 ± 0.37 m/a) for a nearly contemporaneous period (2000–2013).

**Figure 2:**
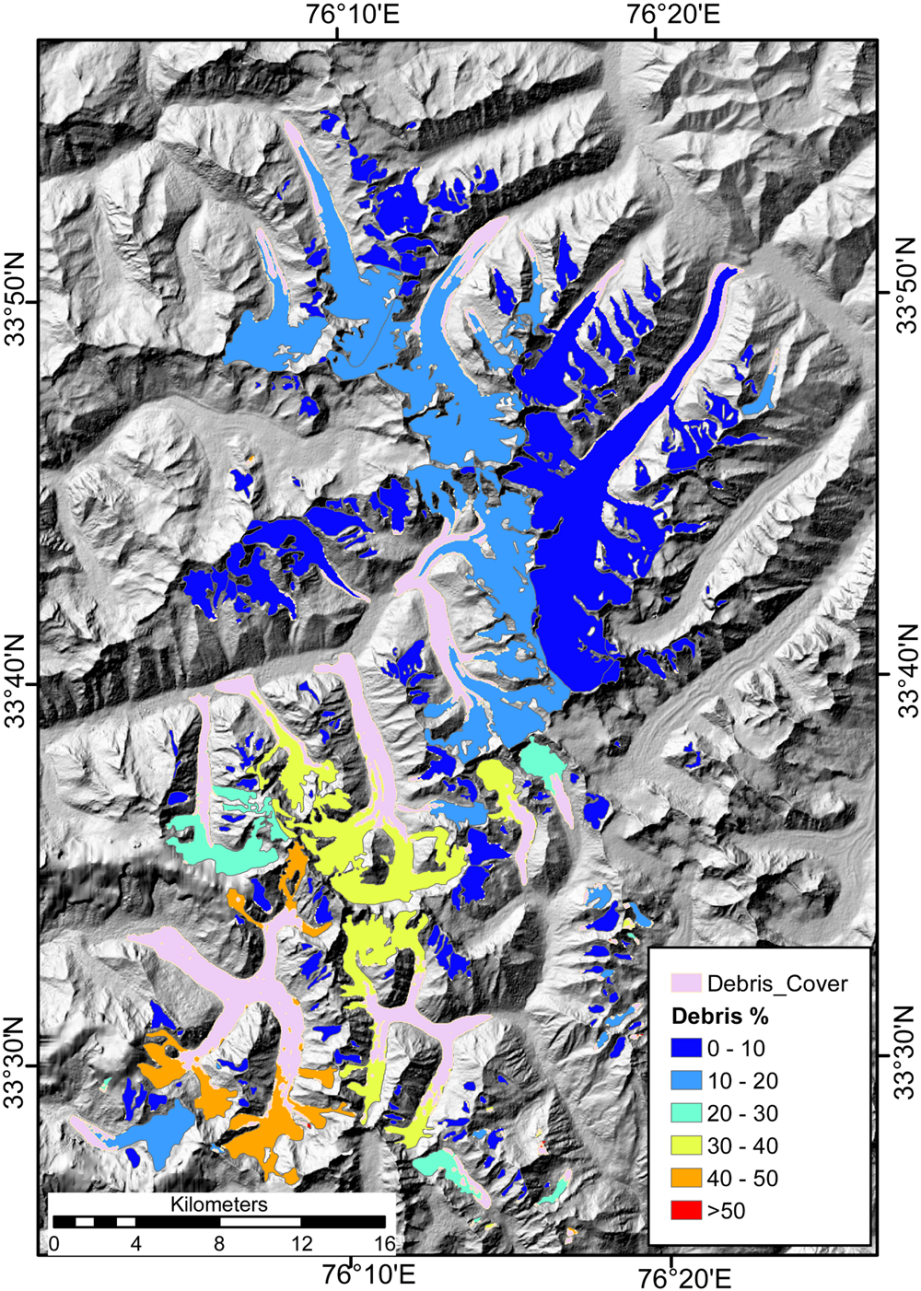
Choropleth map of glacier polygons depicting debris cover percentage with respect to total area overlaid on a hillshade map generated from SRTM DEM. Overlaid on the choropleth map is the spatial extent of debris cover on the glaciers within the study area (light pink polygons). (The maps were generated in ArcGis 9.3 Master Lab Kit and postprocessed in Adobe Illustrator Creative Cloud).

**Figure 3:**
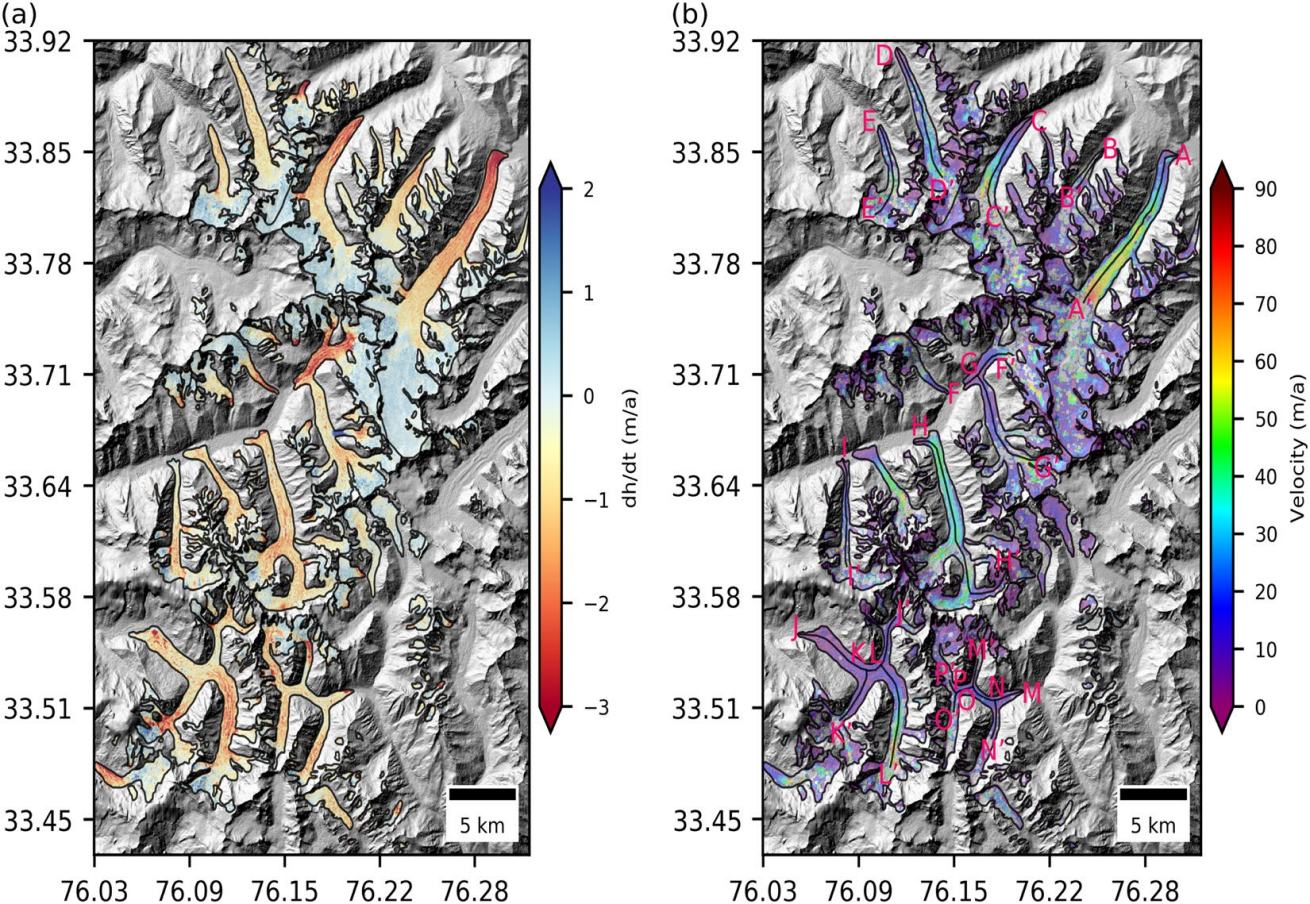
Spatial distribution of elevation change rate (dh/dt) (**a**) and surface velocity (**b**) overlaid on a hillshade map generated from SRTM DEM. The location of profiles to study A-A’ to P-P’ are depicted in (**b**). (The maps were generated in Python (https://www.python.org/), Matplotlib^66^ based image viewer “imviewer” (https://github.com/dshean/imview) and labelling of profile lines was done in Adobe Illustrator Creative Cloud).

**Figure 4:**
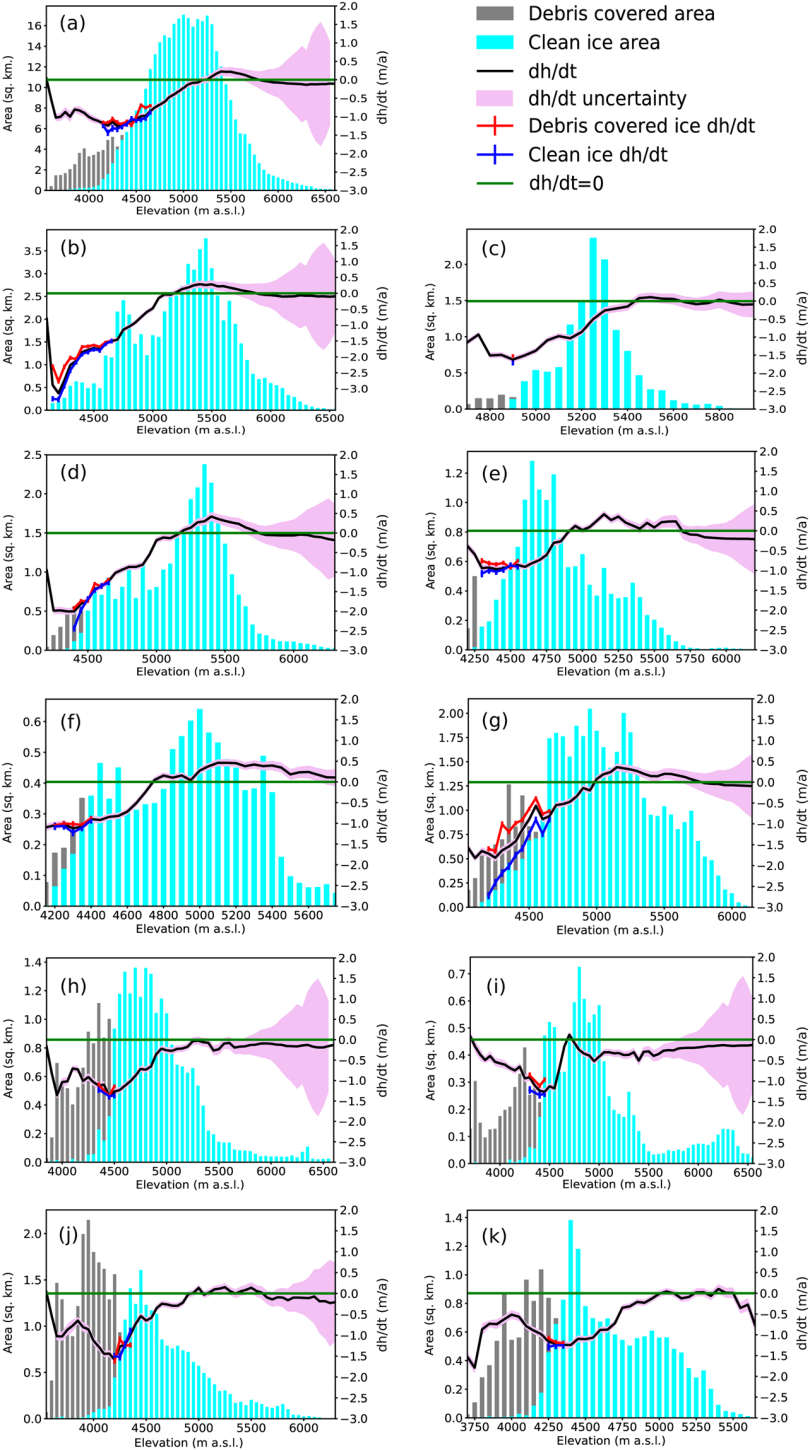
Hypsometric elevation change plots for the (**a**) entire area and for (**b**–**k**) glaciers 1–10 respectively. The blue and grey columns represent total area of clean ice/snow and debris-covered ice respectively, in the corresponding bins. The black line is the median elevation change rate (dh/dt) in each 50 m elevation bin. The red (blue) line is the median elevation change rate for the debris covered ice (clean ice) in the corresponding 50 m elevation bins. The light pink shading along the profile line represents uncertainty in thickness change obtained from equation 5 (Supplementary Section 3). The green line indicates ‘*zero’* dh/dt. (The plots were generated using Python 2.7 (https://www.python.org/), Pandas^67^ and Matplotlib^66^ libraries. The plots were tiled together using Adobe Illustrator Creative Cloud).

The study area consists of glaciers that have different aspect, slope and percentage of debris cover (Fig. 2, Table 1). While some of these glaciers are almost completely debris free (Drung Drung Glacier), others have debris cover percentage as high as ~50% (Boama Glacier) (See Fig. 2). Shown in Fig. 3a,b are elevation change rate (dh/dt) and surface velocity patterns of glaciers in the study area, respectively. In general, most of the glaciers show significant surface lowering in the ablation areas, during the study period (Fig. 3a). However, subtle differences are observed in the elevation change patterns of glaciers with varying coverage of supraglacial debris (Figs 2 and 3a). Similarly, surface velocity patterns of the glaciers reveal an increasing trend with elevation until a maxima is attained near the transient snow lines (Fig. 3b). Supraglacial debris can alter surface albedo and provide insulation, resulting in spatially varying patterns of surface ablation^26^. Therefore, in order to obtain a first order attribution of the variations caused by the presence of debris cover on the mass budget of glaciers, we examine variations in elevation change for the entire study area and 10 major glaciers (occupying ~68% of the total glacierized area) of the study area with respect to their hypsometry (Fig. 4). For all glaciers in the region, the elevation change rate (dh/dt) over debris-covered ice is either less negative or comparable to the rate obtained over clean ice at similar elevations (Fig. 4a). Similar results are obtained from all the individual glaciers as well (Fig. 4b–k), irrespective of the percentage of the debris cover (Fig. 2). However, distinct patterns are observed in the elevation change curves of the individual glaciers (Fig. 4) due to the varying spatial distribution of supraglacial debris (Fig. 2). The observed variation in elevation change curves of individual glaciers is best explained by reduction in debris thickness, and the corresponding insulating effect, with increasing elevation^45–48^. Consequently, the most negative elevation changes occur at the transition zone of debris-covered and clean ice, where the debris thickness is expected to be minimum. Since the transition zone of glaciers with extensive debris coverage throughout their trunk is located at a considerable distance/elevation from the terminus, they experience maximum surface lowering at higher elevations (Figs 2 and 4h–k) as opposed to those which have debris cover limited to their lower tongues only (Figs 2 and 4b–f). The only exception is the Prul Glacier in which the greatest negative elevation change is observed near to its terminus (Fig. 4g) and not at the transition zone between debris-covered and clean ice. This is probably a consequence of the cumulative contribution of two trunks with contrasting elevation change patterns. Prul Glacier has two main trunks lying in a similar elevation range but with varying spatial extent of debris cover. As a result, more negative elevation change values are observed for the south flowing trunk, which has debris cover restricted to the lateral margins only (Fig. 2), compared to the north-flowing trunk that has extensive debris cover (Fig. 3a).

**Table 1:** Details of glaciers with area greater than 10 km^2^ along with their mass budget estimates.

No.	RGI_Id	Name	Area (km^2^)	H I	Debris%	Elevation Range (m.a.s.l.)	Glacier-wide dh/dt (m/a)	Mass Budget (m w.e./a)
1	RGI50–14.18948	Drung Drung	67.58	1.13	6	4088–6544	−0.37 ± 0.13	−0.32 ± 0.11
2	RGI50–14.18909		13.64	1.2	7	4659–5907	−0.63 ± 0.10	−0.53 ± 0.09
3	RGI50–14.18750		33.55	1.16	11	4176–6298	−0.35 ± 0.10	−0.3 ± 0.09
4	RGI50–14.18749		16.6	2.49	11	4154–6174	−0.4 ± 0.08	−0.34 ± 0.07
5	RGI50–14.18755		11.69	1.16	11	4110–5740	−0.15 ± 0.08	−0.13 ± 0.07
6	RGI50–14.18918	Prul	48.18	1.40	19	4002–6113	−0.37 ± 0.10	−0.31 ± 0.08
7	RGI50–14.18964		29.25	2.34	31	3842–6560	−0.75 ± 0.10	−0.64 ± 0.09
8	RGI50–14.18981		13.37	1.78	26	3690–6550	−0.52 ± 0.14	−0.44 ± 0.13
9	RGI50–14.18970	Boama	40.89	2.87	50	3513–6292	−0.77 ± 0.09	−0.66 ± 0.09
10	RGI50–14.17250		23.19	1.69	37	3665–5610	−0.75 ± 0.08	−0.64 ± 0.08

**Figure 5:**
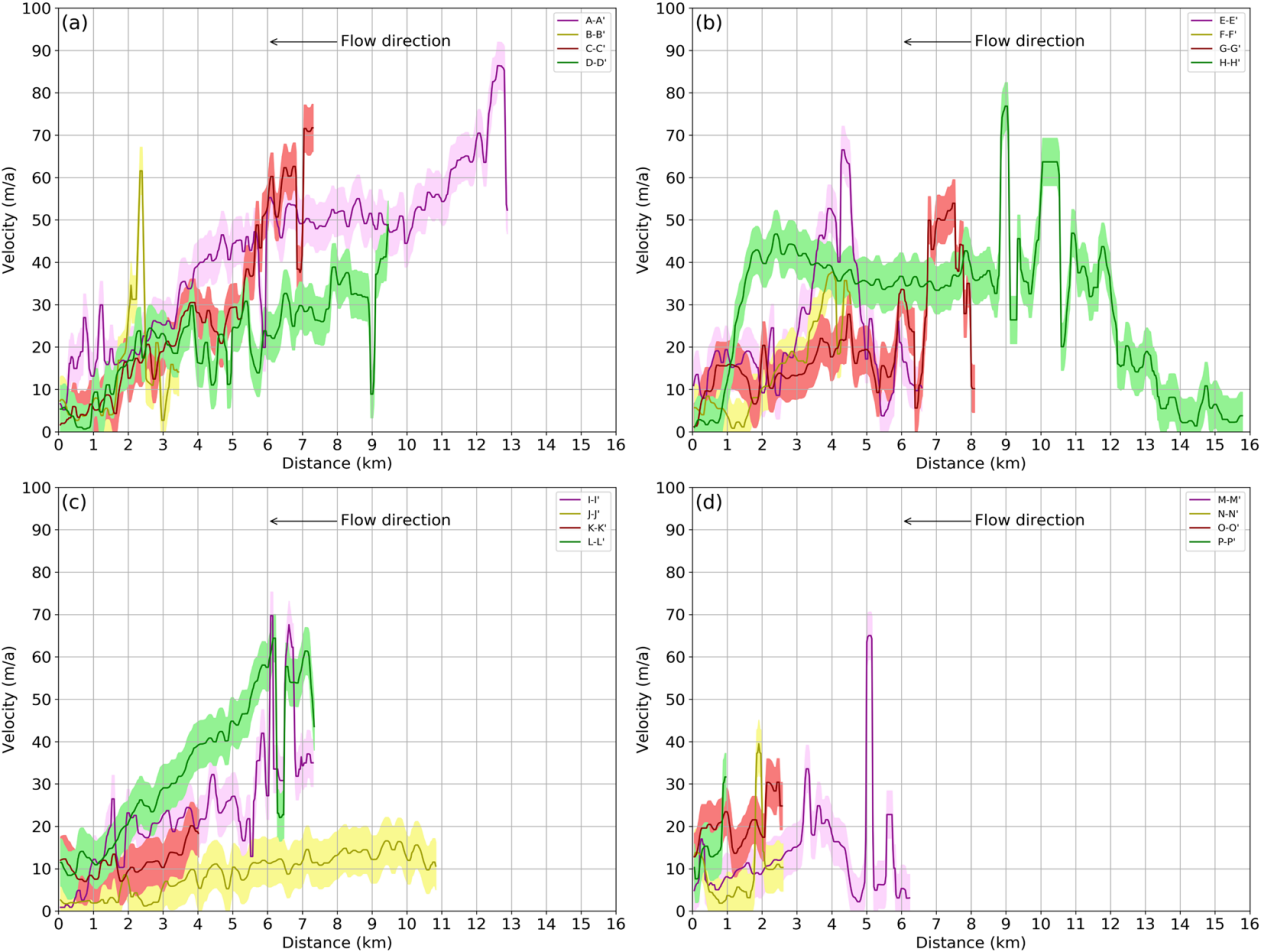
Surface velocity variation along profiles A-A′ to P-P′ for the 2013–14. The light colour shading represents the error in surface velocity estimates (68.3^rd^ percentile, Supplementary Table 2). The profiles have been smoothed using a rolling median filter with a window size of 5 observations (~140 m). Location of the profiles are shown in Fig. 3b. (The plots were generated using Python 2.7 (https://www.python.org/), Pandas^67^ and Matplotlib^66^ libraries).

The spatial patterns in elevation change are also supported by the surface velocity variations (Fig. 5) along the profile lines drawn on the main trunk of glacier (shown in Fig. 3b). The main trunk of Drung Drung glacier is dynamically active throughout, with surface velocities >20 m/a along the main trunk until 0.5 km from the snout (Profile A-A’, Fig. 5a). The surface velocities of glacier number 2–4 are typically >10 m/a along the upstream portions of main trunk until ~2 km from snout, but the surface velocities are <10 m/a along the lower debris covered tongues (Profile B-B’, C-C’, D-D’; Fig. 5a) Interestingly, the tongue of glacier number 5 has surface velocities of 10–20 m/a throughout the length of the profile E-E’ (Fig. 5b), despite it being covered by supraglacial debris, which might be an artefact of persistent discharge into a lake originating very close to the snout (in 2014)^49,50^. High surface velocity near the snout of a lake-terminating glacier is primarily driven by enhanced local ice mass loss due to increase in buoyancy at the calving front^51,52^. Also, there can be changes in basal lubrication due to intrusion of lake water in the glacier sub-system^53^ and changes in surface gradient of glacier^54^, which would result in higher velocity near the snout. Due to the limited coverage of the Carosat-1 DEM the reported elevation change and surface velocity values could not be computed for the final 150 m of the glacier number 5, which is attached to a lake. Recently, the development of a shallow lake has also been reported near the snout of the Drung Drung Glacier^55^ which could also be one of the possible reasons for the sustained mobility of this particular glacier.

Although Prul Glacier has two trunks with varying spatial coverage of debris (Fig. 2), the upstream portion of both trunks demonstrate an active glacier flow until 2 km from the snout after which the surface velocities become less than 10 m/a (Profiles F-F’, G- G’; Fig. 5b). The north flowing glaciers 7 and 8 also demonstrate surface velocities >40 m/a and 20 m/a respectively along the upstream portions of their respective main trunk until ~1.5 km from the snout (Profile H-H’, Fig. 5b; Profile I-I’, Fig. 5c). Only the lower extent of the glaciers, ~1 km near the snout, are having velocities <10 m/a (Fig. 3a). The observed variation in slope is limited and is similar for all the ten major glaciers (Supplementary Fig. S4), which rules out the influence of surface slope in the differential surface dynamics of glacier number 7 and 8. Surface velocity patterns along the main trunk of Boama Glacier (glacier number 9) (Profile J-J’, Fig. 5c) and glacier number 10 (Profile M-M’, Fig. 5d) are similar to those of Prul and other debris covered glaciers in this area, with prominent stagnation near the snout. The glacier stagnancy is most pronounced in the trunk of Boama Glacier which has surface velocities lower than 10 m/a until ~5 km from the snout (Profile J-J’, Fig. 5c). However, the individual tributaries of Boama Glacier and glacier number 10 have surface velocities in excess of 10 m/a along their respective trunks (K-K’, L-L’, N-N’, O-O’ and P-P’; Fig. 5c,d) at increasing distances from their confluence with the main trunk.

This analysis reveals that all the glaciers, irrespective of the presence/absence of debris cover, are dynamically active for at least some portions along their trunk but their debris covered snouts are stagnant. Nonetheless, the relative extent of stagnancy is variable from glacier to glacier. Quincey *et al*.^56^ found a strong relationship between the relative percentage area of stagnancy and the catchment topography of the glaciers in question. In general, debris-covered glaciers, which had considerable percentage of area above a cut-off elevation (~6500 m a.s.l. in the area surrounding Mt. Everest) had higher surface velocities than those whose accumulation area distribution was more skewed towards lower elevation ranges. This was due to increased deposition of snowfall on glaciers with extensive accumulation areas at higher elevations, resulting in an increased amount of available mass flow for transport. In this study we replicate this analysis by considering the hypsometric index of these glaciers, assuming that higher hypsometric index indirectly signifies less ice mass at higher elevations. We find that the hypsometric indices do not have a direct relationship with the extent of glaciers having surface velocities >10 m/a. For example, the hypsometric index for glacier number 7 and 8 (which have surface velocity <10 m/a for only the lower 1 km extent near their snout) are 2.34 and 1.78 respectively, indicating bottom heavy geometry (HI >1.2), while glaciers numbers 6, 9 and 10 (which have surface velocity <10 m/a for the extent >2 km from their respective snouts) have hypsometric indices of 1.40, 2.87 and 1.69 respectively. At the same time, Benn *et al*.^57^ reported that in Nepal Himalaya, stagnancy in debris-covered trunks upstream of the snout is an artefact of local reduction in ice thickness and surface gradient. Since debris-covered glaciers register maximum surface lowering several km upstream from the snout (See Fig. 5 in Benn *et al*.^57^), a local inversion in surface gradient occurs, which result in stagnant low sloping downstream tongues^57^. However, in spite of similar elevation change pattern (maximum surface lowering at higher elevations from the snout) for the ~15 year study period, glacier number 7 and 8 are observed to have consistently high surface velocities over their debris-covered trunks. The strikingly active flow in the lower reaches of glacier number 7 and 8 might also be governed by sustained ice or snow avalanches^22^, differences in subglacial sliding and hydrology in the terminus region^58^ or differences in bedrock properties^42^. But, evaluating the contribution of these individual components is beyond the scope of this study.

As interpreted from the elevation change curves for the glaciers in this study, the rates of surface lowering of debris-covered ice are either lower or comparable to that of clean ice at similar elevations. Such an effect should have translated into a less negative specific mass budget for the glaciers having extensive coverage of supraglacial debris. On the contrary, the mass loss is higher for the most extensively debris-covered glaciers (glaciers with prominent supraglacial debris coverage throughout their main trunk, e.g., glacier number 7, 8, 9 and 10) (Fig. 2 and Table 1). We attribute this to the variation in overall hypsometry of these glaciers, the presence of supraglacial melt ponds/ice cliffs and to the possible difference in overall climate due to the presence of a topographic divide running NW to SE (Fig. 1). Reduced surface melting due to the extensive debris coverage allows these glacier to extend to comparatively lower elevation ranges than clean ice glaciers^48^. Moreover, these extensively debris covered glaciers are predominantly bottom heavy with HI >1.5 (Table 1). This signifies that the distribution of ice mass is more skewed towards lower elevation ranges resulting in a comparatively lower accumulation area ratio. Similar observations were also made for glaciers in Bhutan Himalayas by Maurer *et al*.^59^. Therefore, in spite of the less negative elevation change over debris-covered ice, compared to clean ice at similar elevation (Fig. 3a), smaller magnitudes of negative elevation change integrated over a larger ablation area (Fig. 4 b–k) results in more negative glacier-wide mass budget of the bottom heavy debris covered glaciers than the predominantly clean ice glaciers which generally have a more equi-dimensional or top heavy geometry (Table 1). This observation is further supported by statistically significant influence of hypsometric index and minimum elevation on the percentage of supraglacial debris and specific mass budget of glaciers in the study area (See Supplementary Material: Section 4, Fig. S5). It is further evident that the percentage of debris cover is higher for low sloping glaciers that have a bottom heavy geometry (See Supplementary Material: Section 4, Fig. S5).

The presence of supraglacial ice cliffs and melt ponds also play an important role in enhanced mass loss of debris-covered glaciers^2,22^. Even though the exposed ice cliffs occupy only a small percentage of the total debris covered ablation area, they significantly increase the overall melt rates of the entire debris-covered areas^60–62^. Similarly, melt ponds also have been reported to increase the overall melting of debris-covered glaciers in the Nepal Himalaya^22,45,57^. The higher melt rates associated with ice cliffs and melt ponds is attributed to the preferential absorption of the longwave solar radiation^59^ which is the most important component of solar radiation affecting glacier melting^63^. We analyse modern day high-resolution imagery in Google Earth to quantify the frequency and distribution of ice cliffs and melt ponds for the 10 glaciers in this study. We find that their spatial distribution corresponds with the location of ice stagnancy of the corresponding debris covered zones (Supplementary Fig. S6). For instance, the dynamically decayed main trunk of Boama Glacier has a number of ice cliffs and melt ponds close to each other (Figs 3b and 5c and Supplementary Fig. S6i). However, the coverage of ice cliffs and melt ponds is more sparse in the dynamic trunks of glacier number 7 and 8 (Supplementary Fig. S6g and h). The observed spatial elevation difference and ice dynamics pattern of Boama Glacier bears close resemblance to the pattern reported for Ngozumpa Glacier in Nepal Himalaya, which is extensively covered by melt ponds and cliffs^21,64^. Interestingly, it has been reported earlier that the pronounced stagnation of debris-covered ice tongues coupled with low surface gradient may also enhance the growth rates of the existing melt ponds in the future^10,64^.

A third possible explanation for the higher mass loss of the extensively debris covered glaciers in the region might be the difference in climate setting induced by the presence of the topographic divide running from NW to SE (Fig. 1). Glaciers on the northern side of the divide lie in the rain shadow area of Indian Summer Monsoon (ISM)^65^ and they receive precipitation mainly in the form of snowfall resulting from the MLW during the Northern Hemisphere (NH) winter^28^. In contrast, the glaciers on the southern side of the divide are mostly influenced by ISM and less by MLW. As precipitation from the MLW occurs in NH winter in the form of snowfall, the chances of accumulation and preservation of the incoming snowfall for a longer time are higher. Also, the amount of heat supplied by snow precipitation from the MLW is drastically lower than that supplied by direct rainfall occurring from ISM. Therefore, glaciers lying on the northern side of the divide have a larger accumulation area than those lying to the south, thereby resulting in a comparatively less negative specific mass budget. This result is indirectly supported by our elevation change measurements, which shows that significant portions of the glaciers lying on the northern side of the divide are either having positive or near zero elevation change at high elevations (Figs 3a,4b–e)). The Prul Glacier (glacier number 6), which lies almost on the divide also shows similar positive elevation change at higher attitudes (Figs 3b and 4g). However, the trend is absent for glaciers lying further south (Figs 3b and 4h–k), hinting at the decreasing influence of MLW. This proposed assertion is based only on the established theories of precipitation for the study area since we do not have actual weather station data from either sides of the divide to fully support our claims. Thus, further detailed meteorological studies needs to be carried out to explore the influence of precipitation in driving these changes.

## Conclusion

A detailed mass budget and surface velocity assessment of glaciers is important for characterizing heterogeneity in glacier dynamics and for assessing the control of local scale factors such as variations in topography, slope, aspect, debris cover and hypsometry in influencing this heterogeneity. Consequently, these estimates allow for fine tuning glacier-wide energy and surface mass balance models which are in turn important for calibrating large scale hydrological models and assessing the contribution of melt water to river discharge. In this study, we estimate mass budget and surface velocity for a glacierized region covering an area of ~437 km^2^ in the Zanskar Basin of Western Himalaya. Results reveal prevalent mass loss of the glaciers in the Zanskar Basin and characterize the heterogeneity in elevation change/surface velocity patterns amongst the different types of glaciers based on their debris-cover extent, hypsometry and orientation. The overall elevation change rate obtained for glaciers in the study area is −0.45 ± 0.1 m/a, amounting to a specific mass budget of −0.38 ± 0.09 m w.e./a. Comparison of elevation change rates at similar elevation bins reveal less negative or almost similar values over debris-covered ice in comparison to clean ice. However, the amount of ice mass loss for extensively debris-covered glaciers is higher than that of relatively clean-ice glaciers. This is despite our analysis suggesting strong insulating effect of debris cover against surface melting. We attribute the mass budget variations primarily to the bottom heavy geometry of certain glaciers and due to the effect of enhanced melting caused by the presence of ice cliffs and melt ponds on the surface of extensively debris-covered glaciers. Also presented are evidences of the role of variations in the precipitation system across a topographic divide, ISM based hydroclimatology as opposed to MLW, in controlling the mass budget of specific glaciers.

This study also highlights important differences in the characteristics of clean and debris-covered glaciers that are crucial for a detailed understanding of regional glacier dynamics. For example, significant variations in the elevation change pattern of clean and extensively debris-covered glaciers are observed. Negative elevation change is maximum near the snout of the glaciers which are relatively free of debris, while the same occurs several kilometres upstream in the case of extensively debris-covered glaciers. Similarly, clean-ice glaciers maintained considerable surface velocity throughout their main trunk but debris-covered glaciers exhibited marked reduction in surface velocity near their debris covered tongues. This is probably an artefact of the reduction in driving stresses caused by increased insulation provided by debris cover. Interestingly two extensively debris covered glaciers depicted consistent mobility almost throughout their entire trunk. Overall, results presented in this study underscore the variability in the characteristics of glaciers even within a single basin and reinstates the need for detailed assessment of glacier dynamics and mass budget over a smaller spatial domain.

## Electronic supplementary material

Supplementary Material
