# Supplementary material for: Assessing controls on mass budget and surface velocity variations of glaciers in Western Himalaya
Source: Sci Rep. 2018 Jun 11;8:8885. doi: 10.1038/s41598-018-27014-y (PMC5996025; doi:10.1038/s41598-018-27014-y)
Supplement: Supplementary file 1 — Supplementary Material [file 41598_2018_27014_MOESM1_ESM.pdf]

SUPPLEMENTARY MATERIAL

Assessing controls on mass budget and surface velocity variations of glaciers in Western Himalaya

[1]Shashank Bhushan, [1*]Tajdarul H. Syed, [2,3]Anthony Arendt, [4]Anil V. Kulkarni, and [1]Debanjan Sinha
[1]Department of Applied Geology, Indian Institute of Technology (ISM), Dhanbad, India.
[2]Applied Physics Laboratory, University of Washington, Seattle, U.S.A.
[3]eScience Institute, University of Washington, Seattle, U.S.A.
[4]Divecha Centre for Climate Change, Indian Institute of Science, Bengaluru, India
* Corresponding Author (Email ID:tsyed@iitism.ac.in)

1) Coregistration of DEMs:

Because we do not use any ground control points (GCPs) in deriving our Cartosat-1 DEM, proper coregisteration and alignment of the DEMs with respect to a valid reference is very crucial for achieving accurate mass budget estimates and proper quantification of uncertainty. Hence we coregister the Cartosat-1 DEMs to the reference DEM (SRTM). For this, the two Cartosat-1 DEMs are resampled to 30 m resolution using the bilinear technique and the SRTM DEM is reprojected to UTM 43N coordinates at a posting of 30 m to make both the DEMs conformable to each other. The SRTM DEM is subtracted from the more recent Cartosat-1 DEMs. Horizontal coregisteration is achieved by computing and adjusting for the mean shift, which is obtained by minimizing the standard deviation of elevation differences on glacier free terrain[1]. The resultant DEMs are further coregistered by iteratively adjusting the offsets over stable terrain derived from the analytical relationship between elevation change, slope and aspect[2] (Equation 1). For determining the offsets, only pixels having slopes less than 30º are considered[3]. The process is repeated until the shifts in X, Y and Z direction are less than 1 m. Subsequently, the

observed trends in the obtained elevation change map (Cartosat-1 minus SRTM) are corrected on the basis of a second order trend surface constructed from stable (ice-free) area pixels having slopes less than 15°[3]. The obtained elevation change maps are corrected for remaining median offset on stable terrain andare then mosaiced using *''median'* technique for further analysis.

$$dh = a.\tan\alpha.\cos(b-\varphi) + dh' \qquad \text{(Equation 1)}$$

where, dh, α, and Ψ represent elevation change at individual pixels, terrain slope and aspect respectively. The terms *a*, *b*, dh' denote the magnitude of horizontal shift, direction of the shift vector, and overall elevation bias between two DEMs respectively.

2) Gap filling of outliers:

Generally, downwasting is expected to be highest near the snout and lowest in the accumulation region. Thus, it is not suitable to use the same threshold for limiting the outliers in both the accumulation and ablation region[4]. Keeping this in mind, outliers over the glacierized region are defined as per an elevation dependent 'non-linear' sigmoidal relationship (Equation 2)[3–5]. All data gaps (due to voids in the DEMs and those created after outlier filtering) are filled up using ordinary kriging, assuming an isotropic distribution of data. This elevation dependent outlier detection technique is able to remove elevation change values at higher elevations, with very steep slopes (>30°), since the accuracy of the DEM over these regions is questionable.

$$\Delta h_{\max} = \left[5 - 5\tanh\left\{2\pi - 9\left(\frac{E_{MAX} - E_{MIN}}{E_{GLACIER}}\right)\right\}\right] * STD_{GLACIER} \qquad \text{(Equation 2)}$$

where $\Delta h_{\max}$ is the maximum allowable elevation change for the particular pixel, E_{MAX} is the maximum glacier elevation in the watershed, E_{MIN} is the minimum glacier elevation in the watershed, $E_{GLACIER}$ is the elevation of the particular pixel, $STD_{GLACIER}$ is the unfiltered standard deviation of the elevation change in the glacierized area in the entire study area. The initial, outlier removed and gap filled elevation change maps are shown in Figure S1.

3) Penetration Correction and Uncertainty Estimation

In order to correct the bias introduced due to the penetration of C Band signal into snow and ice, we use average C Band penetration values of 2.3±0.9 m (1.7±0.7 m) for snow/firn (clean ice) given by Kääb et al.[6] and assume the penetration in debris-covered parts of the glacier to be zero. Debris covered area is distinguished from clean ice/snow areas using a threshold of 2.2 on the band ratio of Red to Short Wave Infrared (SWIR)[6]. Isolated debris-covered pixels are removed by employing a series of erode and dilate filters with a conservative, 2 iterations, approach. Clean ice is separated from firn/snow by a threshold DN value of 125 on the digital image of the panchromatic band of Landsat 7 ETM+[7].

Uncertainty in elevation changes are quantified using the approach suggested by Gardelle et al.[8] (Equation 3 and 4).

$$\sigma = \frac{E_{\Delta h_i}}{\sqrt{N_{eff}}} \qquad \text{(Equation 3)}$$

$$N_{eff} = \frac{N_{tot} * R}{2d} \qquad \text{(Equation 4)}$$

$E_{\Delta h_i}$ is the standard deviation of the mean elevation change of non-glacierized terrain per 50 m elevation bin, and N_{eff} is the effective and N_{tot} the total number of observations. R is the pixel size (30 m) and d is the distance of spatial autocorrelation. d is equal to the range of influence of the obtained modelled spherical semi-variogram for elevation change values on stable terrain and is equal to ~475 m[9,10]. To quantify the uncertainty in elevation change over glacierized area spanning several elevation bins, weighted averages of σ with respect to glacier hypsometry are calculated. Thus, each glacier has a different elevation change uncertainty and the uncertainty is also different for the entire basin which depends on the hypsometric distribution of the glacier in question.

The overall uncertainty in thickness change (contributed due to uncertainty in elevation changes and C Band penetration) (U_{DTM}) and mass budget (U_M)[5] will be quantified by (5) and (6) respectively.

$$U_{DTM} = \sqrt{(\sigma)^2 + (\Delta p)^2} \quad \text{(Equation 5)}$$

$$U_M = \sqrt{\left(\frac{\Delta h * \Delta \rho}{t * \rho_w}\right)^2 + \left(\frac{U_{DTM} * \rho_i}{t * \rho_w}\right)^2} \quad \text{(Equation 6)}$$

where, σ is the relative vertical accuracy obtained by equation 3, Δp is the uncertainty in C Band radar penetration correction (0.9 m), Δh is the observed thickness change for the glacier area, t is the time period of observation, $\Delta \rho$ is the uncertainty in density estimate (60 kg/m^3)[11], ρ_w is the density of water (1000 kg/m^3), ρ_i is the density value used for ice/snow (850 kg/m^3).

We do not apply any seasonality correction as both of our Cartosat-I scenes were obtained at the end of ablation season but assume that the effect is within the uncertainty estimates obtained.

4) Statistical Analysis of relationship between different parameters with potential influence on glacier-wide mass budget

In order to determine the relationship between mass budget and different parameters such as slope, aspect, area, glacier-wide mass budget, hypsometric index (HI), debris-percentage and elevation distribution, scatter plots for glaciers greater than 1 km^2 (number of glaciers =43) are constructed (Figure S5). The respective correlation scores/significance values are calculated using the Kendall-tau test for each combination. Smaller glaciers spanning a limited number of elevation bands and terminating at higher elevations generally have less negative glacier-wide mass budget (indicated by the cluster near small area values; Figure S5a). This clustering translates into a negative correlation with a significant score, otherwise glacier-wide mass budget is generally independent of the total area of the glaciers. Glacier-wide mass budget of glaciers with low median slopes is observed to be more negative than those with high slopes (Figure S5b). Variations in the aspect of glaciers is linearized by calculating the absolute mean deviation from south[12]. No characteristic pattern is observed between glacier-wide mass budget and aspect, which signifies a lower control of aspect on the mass budget of the glaciers. (Figure S5c). Large glaciers (area > ~10 km^2) extending to lower elevation ranges are observed to have a more negative glacier-wide mass budget (Figure S5d). Glacier-wide mass budget is generally independent of the maximum elevation of glacier extent (Figure S5e). Glacier-wide mass budget is more negative for larger glaciers with lower median and minimum elevation (Figure S5f). Glacier-wide mass budget of glaciers with *top heavy*

or *equi-dimensional* geometry is generally less negative, however glacier-wide mass budget is more negative for glaciers with *bottom heavy* geometry, terminating at lower elevations (Figure S5g). Debris percentage is higher for *bottom heavy* glaciers with lower median slopes (Figure S5h). Glacier-wide mass budget is more negative for glaciers with a higher percentage of debris (Figure S5i). However, as concluded from Figure S5d,j,h & j, the observed correspondence has more to do with the relationship of slope, elevation range and hypsometric index with glacier-wide mass budget than debris percentage independently. From the above analysis, we can conclude that the debris percentage is affected by the hypsometry and slope distribution of glaciers. Generally, debris-cover percentage being higher for low sloping *bottom heavy* glaciers. Bottom heavy glaciers with low median slopes allow the build-up of debris cover over time as the debris falls from the steep accumulation zones and surrounding headwalls and settles onto the low sloping tongues. Glacier-wide mass budget of low sloping *bottom heavy* glaciers located in lower elevation zones is generally more negative. The extensive coverage of supraglacial debris on the bottom heavy glaciers allow them to terminate at much lower elevations, an observation which is also supported by an earlier modelling study[13] as well.

Reference:

1. Berthier, E. *et al.* Remote sensing estimates of glacier mass balances in the Himachal Pradesh (Western Himalaya, India). *Remote Sens. Environ.* **108,** 327–338 (2007).

2. Nuth, C. & Kääb, A. Co-registration and bias corrections of satellite elevation data sets for quantifying glacier thickness change. *The Cryosphere* **5,** 271–290 (2011).

3. Goerlich, F., Bolch, T., Mukherjee, K. & Pieczonka, T. Glacier Mass Loss during the 1960s and 1970s in the Ak-Shirak Range (Kyrgyzstan) from Multiple Stereoscopic Corona and Hexagon Imagery. *Remote Sens.* **9,** 275 (2017).

4. Bhattacharya, A. *et al.* Overall recession and mass budget of Gangotri Glacier, Garhwal Himalayas, from 1965 to 2015 using remote sensing data. *J. Glaciol.* **62,** 1115–1133 (2016).

5. Pieczonka, T. & Bolch, T. Region-wide glacier mass budgets and area changes for the Central Tien Shan between ~1975 and 1999 using Hexagon KH-9 imagery. *Glob. Planet. Change* **128,** 1–13 (2015).

6. Kääb, A., Berthier, E., Nuth, C., Gardelle, J. & Arnaud, Y. Contrasting patterns of early twenty-first-century glacier mass change in the Himalayas. *Nature* **488,** 495–498 (2012).

7. Jiskoot, H., Curran, C. J., Tessler, D. L. & Shenton, L. R. Changes in Clemenceau Icefield and Chaba Group glaciers, Canada, related to hypsometry, tributary detachment, length–slope and area–aspect relations. *Ann. Glaciol.* **50,** 133–143 (2009).

8. Gardelle, J., Berthier, E., Arnaud, Y. & Kääb, A. Region-wide glacier mass balances over the Pamir-Karakoram-Himalaya during 1999-2011. *The Cryosphere* **7,** 1263–1286 (2013).

9. Wang, D. & Kääb, A. Modeling Glacier Elevation Change from DEM Time Series. *Remote Sens.* **7,** 10117–10142 (2015).

10. Magnússon, E., Muñoz-Cobo Belart, J., Pálsson, F., Ágústsson, H. & Crochet, P. Geodetic mass balance record with rigorous uncertainty estimates deduced from aerial photographs and lidar data – Case study from Drangajökull ice cap, NW Iceland. *The Cryosphere* **10,** 159–177 (2016).

11. Huss, M. Density assumptions for converting geodetic glacier volume change to mass change. *The Cryosphere* **7,** 877–887 (2013).

12. Salerno, F. *et al.* Debris-covered glacier anomaly? Morphological factors controlling changes in the mass balance, surface area, terminus position, and snow line altitude of Himalayan glaciers. *Earth Planet. Sci. Lett.* **471,** 19–31 (2017).

13. Rowan, A. V., Egholm, D. L., Quincey, D. J. & Glasser, N. F. Modelling the feedbacks between mass balance, ice flow and debris transport to predict the response to climate change of debris-covered glaciers in the Himalaya. *Earth Planet. Sci. Lett.* **430,** 427–438 (2015).

14. Hunter, J. D. Matplotlib: A 2D Graphics Environment. *Comput. Sci. Eng.* **9,** 90–95 (2007).

15. Travis, E. Oliphant. A guide to NumPy, USA: Trelgol Publishing, (2006)

16. McKinney, W. Data Structures for Statistical Computing in Python, *Proceedings of the 9th Python in Science Conference*, 51-56 (2010).

17. Jones, E., Oliphant, E, Peterson, P., *et al.* SciPy: Open Source Scientific Tools for Python, 2001-, http://www.scipy.org/ [Online; accessed 2018-05-02]

Table S1 : Statistics of elevation change values (Cartosat-1 – SRTM) over stable ground.

After Planametric Correction	Mean/Median bias (m)	Standard deviation (m)	NMAD (m)	68.3rd percentile (m)	Maximum/Minimum (m)
Original	0.77/0.44	14.91	8.37	**5.74**	-157.88/146.36
Outlier removed*	0.78/0.43	10.86	8.16	**4.40**	-43.95/45.49

*Outliers are defined as values outside the mean +/- 3 standard deviation limit over stable areas.

Table S2:- Statistics of velocity values over stable terrain

Mean/Median (m/a)	Standard Deviation (m/a)	NMAD (m/a)	68.3rd Percentile (m/a)	Maximum/Minimum (m/a)
8.56/3.62	14.71	2.81	**5.58**	0.00/102.67

Supplementary Figures

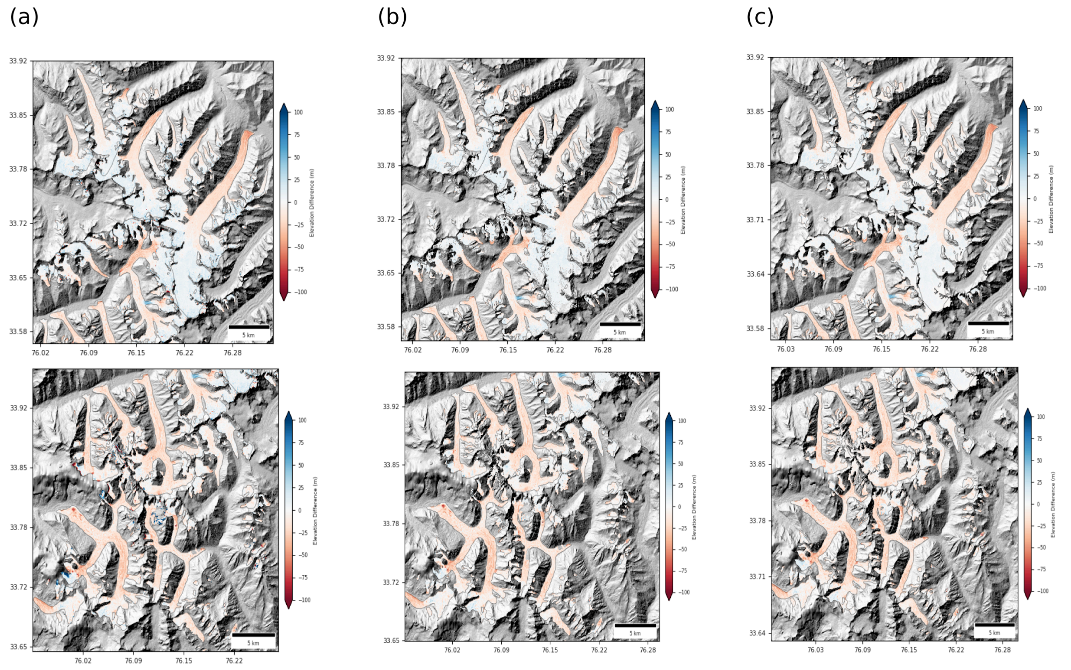

Figure S1: Elevation change map of glacerized area after (a) planimetric correction, (b) outlier filtering and (c) gap filling and penetration correction. (The maps were generated in Python (https://www.python.org/) Matplotlib[14] based image viewer "imviewer" (https://github.com/dshean/imview). The image was tiled together using Adobe Illustrator Creative Cloud).

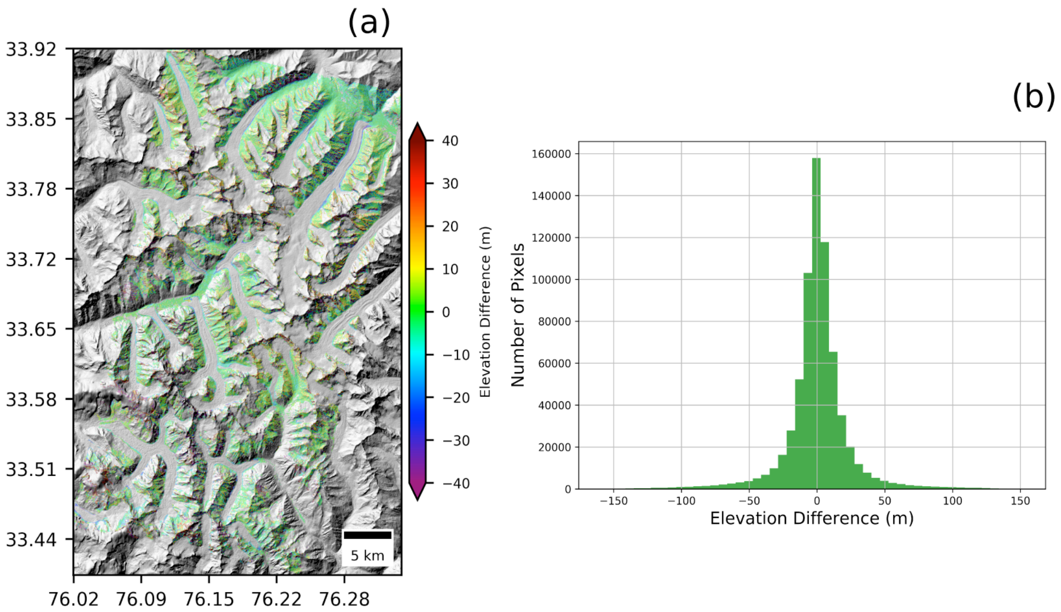

Figure S2: (a) Elevation change map and (b) histogram distribution of elevation change over non-glacierized stable terrain. (The map was generated in Python Matplotlib[14] based image viewer "imviewer" (https://github.com/dshean/imview) and the (b) plot was generated using Python 2.7 (https://www.python.org/) NumPy[15] and Matplotlib[14]. The image was tiled together using Adobe Illustrator Creative Cloud).

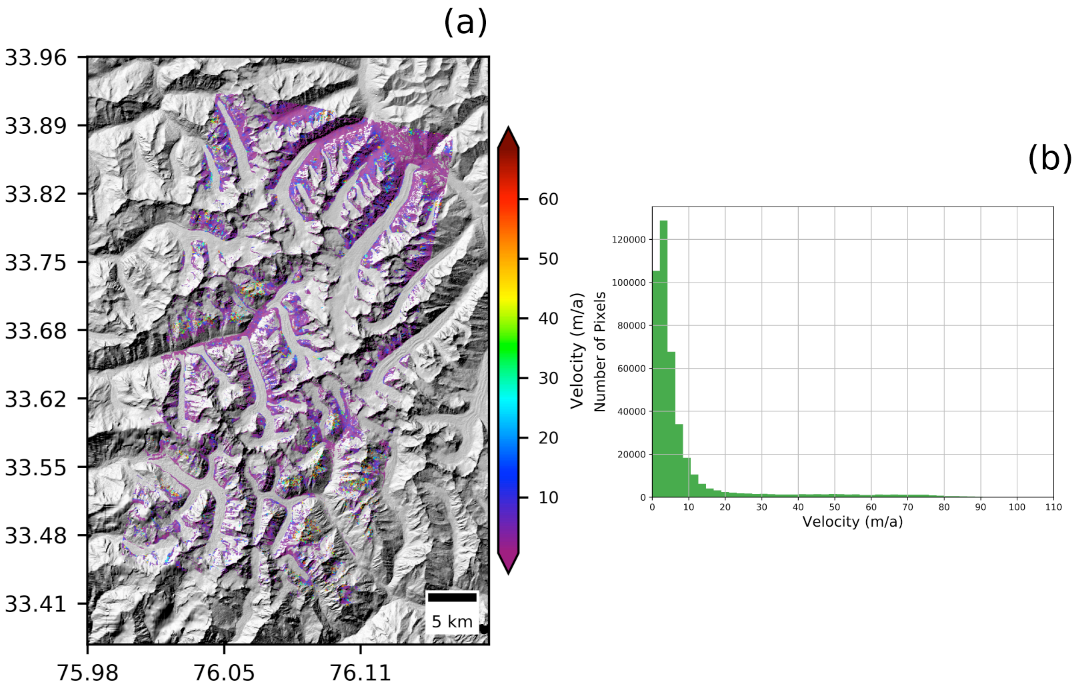

Figure S3: (a) Surface velocity map and (b) histogram distribution of surface velocity over non-glacierized stable terrain. (The map was generated in Python

(https://www.python.org/) Matplotlib[14] based image viewer “imviewer” (https://github.com/dshean/imview) and the (b) plot was generated using Python 2.7 (https://www.python.org/) NumPy[15] and Matplotlib[14]. The image was tiled together using Adobe Illustrator Creative Cloud).

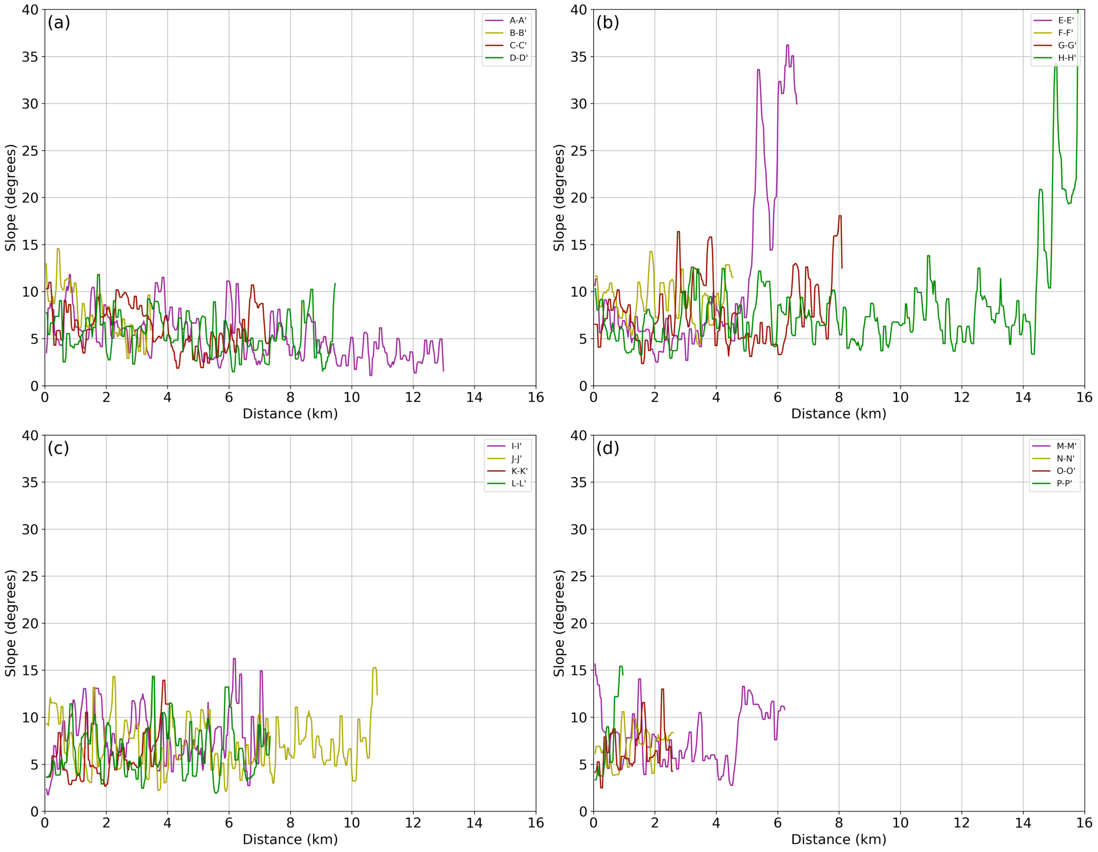

Figure S4: Slope variation along profiles A-A′ to P-P′ computed from SRTM DEM. The profiles have been smoothed using a rolling median filter with a window size of 5 observations (~140 m). The location of the profile lines are shown in Figure 3b. (The plots were generated using Python 2.7 (https://www.python.org/), Pandas[16] and Matplotlib[14] libraries).

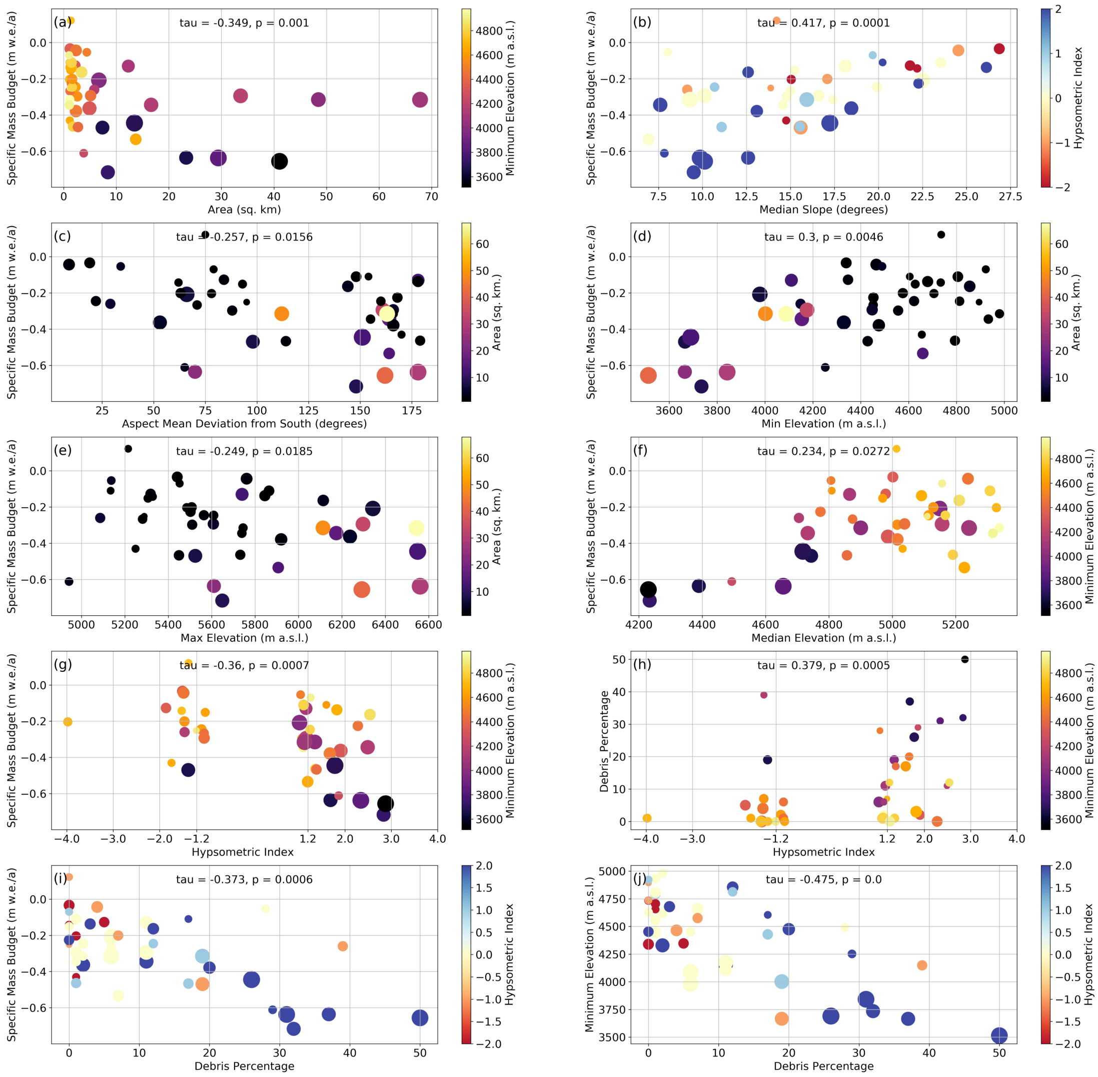

Figure S5: Scatter plot depicting relationships between different glacier properties. The size of the scatter markers are proportional to the number of 50m elevation bins it is comprised of. The only exception is (h), in which the size of scatter markers are proportional to the median slope of the glaciers. (The plots were generated using Python 2.7 (https://www.python.org/), Pandas[16], Scipy[17] and Matplotlib[14] libraries).

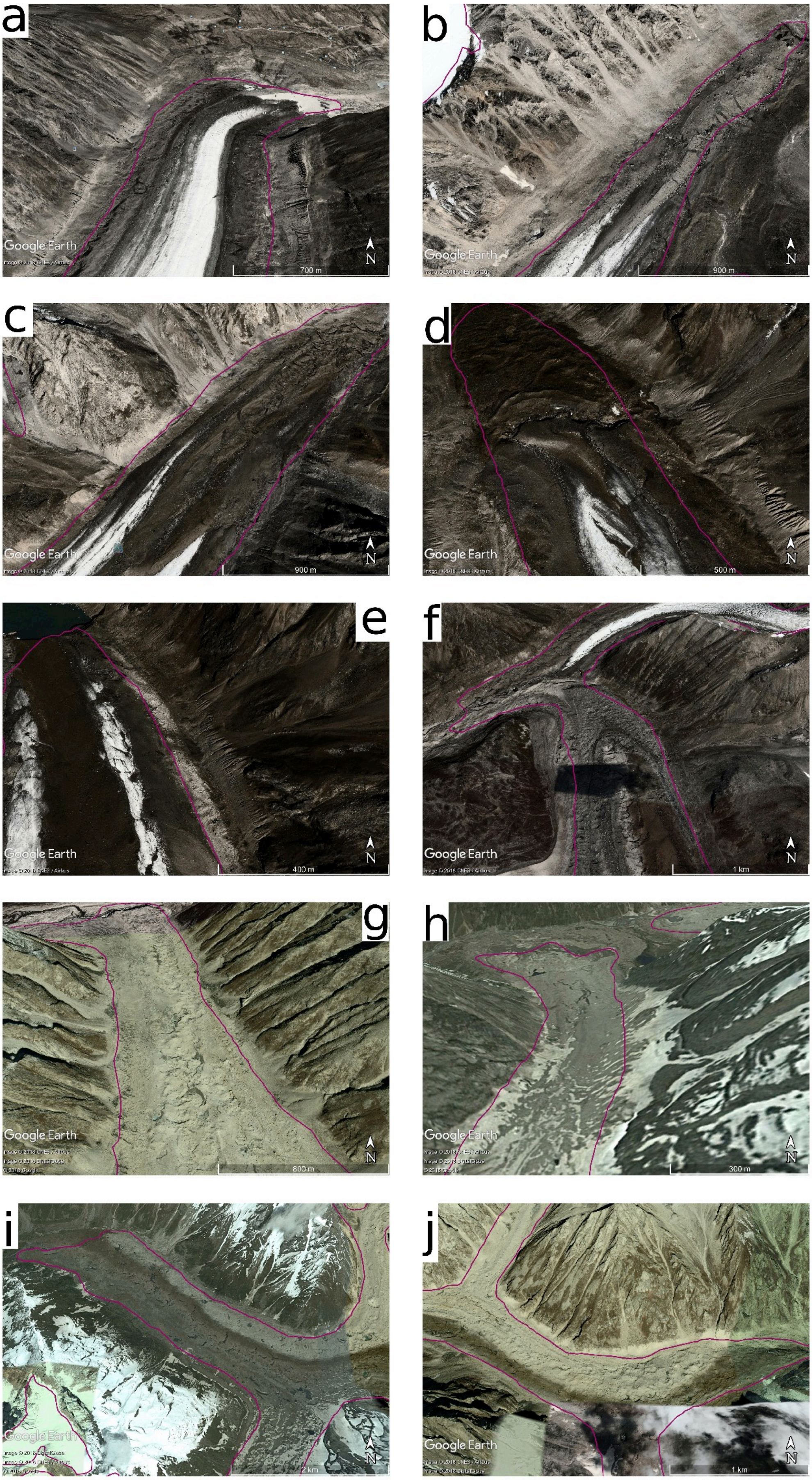
a
Google Earth
N
700 m
b
Google Earth
N
900 m
c
Google Earth
N
900 m
d
Google Earth
N
500 m
e
Google Earth
N
400 m
f
Google Earth
N
1 km
g
Google Earth
N
800 m
h
Google Earth
N
300 m
i
N
2 km
j
Google Earth
N
1 km

Figure S6: Google Earth imagery of the study area depicting debris-covered portions of the ten major glaciers (a to j) considered in this study. The glacier outlines are respresented by the shapefiles used in the geodetic elevation change measurements reproted in the manuscript (The images were obtained from Google Earth (Map data: Google, CNES/Airbus, DigitalGlobe) and were tiled together using GIMP).
